# Pulmonary Tuberculosis in a Patient with COVID-19 Pneumonia

**DOI:** 10.1590/0037-8682-0314-2021

**Published:** 2021-07-02

**Authors:** Cyro Antonio Fonseca, Gláucia Zanetti, Edson Marchiori

**Affiliations:** 1 Hospital Unimed Rio, Rio de Janeiro, RJ, Brasil.; 2 Universidade Federal do Rio de Janeiro, Rio de Janeiro, RJ, Brasil.

A 58-year-old man was admitted to the emergency department with a three-day history of fever, cough, and dyspnea. At admission, he was tachypneic (respiratory rate of 30 breaths/min), his body temperature was 38.2ºC, and his oxygen saturation was 89%. Laboratory findings were unremarkable.

**FIGURE 1:**
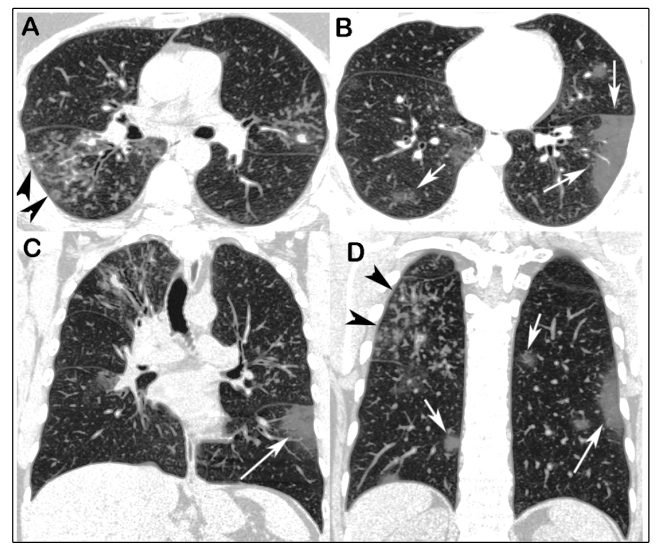
Unenhanced chest CT images with axial (A and B) and coronal (C and D) reconstructions showing “tree-in-bud” opacities predominating in the right lung (black arrowheads), with bronchial wall thickening and small nodules. Note also the multifocal ground-glass opacities in both lungs (white arrows). No pleural effusion or lymph node enlargement was present.

Chest computed tomography showed multiple areas of ground-glass opacity in both lungs, suggestive of viral infection, and tree-in-bud opacities with bronchial wall thickening and small nodules, suggestive of pulmonary tuberculosis (TB; Figure 1). The patient was diagnosed as having COVID-19 by real-time polymerase chain reaction. The diagnosis of TB was confirmed by culture.

The literature on the occurrence of COVID-19 in patients with TB is limited. COVID-19 can occur before, at the time of, or after the diagnosis of TB, and more evidence is required to determine whether it may reactivate or worsen active TB. Data on the association between TB and COVID-19 are not conclusive, but most researchers believe that concurrent infection is likely to worsen TB^1^ ^-^ ^3^.

Careful analysis of the tomographic aspects in such cases can be decisive in gaining clarity about the suspected association between the two diseases. Bilateral ground-glass opacity is the most common pattern in patients with COVID-19. Pulmonary TB patterns include consolidation, cavitary lesions, bronchial wall thickening, and the “tree-in-bud” pattern^1^ ^-^ ^3^.
